# Should a hospitalized child receive empiric treatment with acyclovir?

**DOI:** 10.1186/1824-7288-38-72

**Published:** 2012-12-17

**Authors:** Dina M Kulik, Magda Mekky, Ming Yang, Ari Bitnun, Patricia C Parkin

**Affiliations:** 1 Divisions of Pediatric Medicine and the Pediatric Outcomes Research Team (PORT), University of Toronto, Faculty of Medicine, Toronto, ON, Canada; 2 Division of Infectious Diseases, University of Toronto, Faculty of Medicine, Toronto, ON, Canada; 3 Departments of Pediatrics, University of Toronto, Faculty of Medicine, Toronto, ON, Canada; 4 Health Policy, Management and Evaluation, University of Toronto, Faculty of Medicine, Toronto, ON, Canada; 5 Child Health Evaluative Sciences, Hospital for Sick Children Research Institute, Toronto, ON, Canada; 6 The Hospital for Sick Children, 555 University Ave, Toronto, ON, M5G 1X8, Canada

**Keywords:** Acyclovir, Herpes encephalitis, Herpes simplex, Clinical features

## Abstract

**Background:**

Herpes simplex encephalitis is associated with substantial morbidity and mortality and may be related to timely diagnosis and treatment. While awaiting the results of testing, hospitalization and empiric treatment with acyclovir is recommended, though the direct and indirect costs associated with this management are substantial. We sought to examine children hospitalized for possible herpes simplex encephalitis, following clinical and laboratory assessment in the emergency department, and empiric treatment with acyclovir, in order to describe the proportion receiving a complete course of treatment; and to identify the clinical variables which are associated with receiving a complete course, as compared with an incomplete course of acyclovir.

**Methods:**

Hospitalized children prescribed acyclovir were included in this case control study. Clinical, laboratory and diagnostic variables were abstracted for children prescribed a complete (≥ 14 days) or an incomplete course (<14 days) of acyclovir. Odds ratios and 95% confidence intervals were calculated.

**Results:**

289 children met eligibility criteria, 30 (10%) received a complete course and 259 (90%) received an incomplete course. A history of mucocutaneous herpes simplex virus infection (p < 0.01), Glasgow Coma Scale ≤ 13 (p = 0.02), focal neurologic findings (p = 0.001) and elevated cerebrospinal fluid white blood cell count (p = 0.05) were associated with a complete course of acyclovir.

**Conclusions:**

Many children did not complete a full course of therapy. Unnecessary testing and treatment is burdensome to families and the health care system. Possible predictive variables include abnormal Glascow Coma Scale, focal neurologic findings and cerebrospinal fluid pleocytosis.

## Background

Prospective studies have shown that herpes simplex virus (HSV) accounts for approximately 5% of all cases of acute encephalitis in children [1,2]. Morbidity and mortality for herpes simplex encephalitis (HSE) are significant and may be related to timely diagnosis and treatment [1,3]. Although HSV cerebrospinal fluid polymerase chain reaction (CSF PCR) is considered the test of choice for HSE, it has been shown that the sensitivity in children is lower than in adults, and that a single negative test may later become positive [1,4,5]. Mortality rates for untreated HSE approximate 70% with significant cognitive impairment in those that survive [6]. Therefore, while awaiting the results of testing, hospitalization and empiric treatment with acyclovir is recommended, along with additional investigations such as electroencephalogram (EEG) and neuro-imaging [1,6].

A body of literature has emerged regarding testing for HSV in neonates and young infants less than 3 months of age in Emergency Department (ED) settings [7-11]. These authors have suggested that both the direct and indirect costs of testing are substantial, arguing for the development of a clinical prediction rule to identify low-risk infants [11]. Similar challenges exist in decision making regarding testing and treatment of older children, beyond the neonatal period, presenting with features of a possible central nervous system (CNS) infection. Furthermore, while ED physicians may make initial decisions regarding which children merit lumbar puncture, CSF HSV PCR testing and empiric acyclovir treatment, hospital physicians must determine duration of acyclovir treatment, need for subspecialty consultation and additional investigations, and time of hospital discharge.

The objective of this study was to examine children hospitalized for possible HSE, following clinical and laboratory assessment in the ED, and initiation of empiric treatment with acyclovir, in order to describe the proportion receiving a complete course of treatment; and to identify the clinical variables, obtained within the first 12 hours of assessment, which are associated with the children receiving a complete course of acyclovir, as compared with an incomplete course of acyclovir.

## Methods

This case-control study was conducted on patients admitted to the Pediatric Medicine Inpatient Unit (PMIU) at the Hospital for Sick Children, Toronto, Canada. In our institution, children are initially assessed in the Pediatric Emergency Department, where initial clinical assessment, investigations and empiric therapy is often initiated. Children with suspected HSE who are not immunocompromised and do not require intensive care are admitted to the PMIU and attended by Pediatric Hospitalists. This unit has approximately 4000 admissions per year [12]. The attending Hospitalists are the primary decision makers regarding ongoing therapy.

Since 1994, all children who fulfill previously described stringent criteria (but not all children for whom HSE is initially considered) have been prospectively enrolled in an encephalitis registry [1]. Patients in the registry were considered to have encephalitis if they had depressed or altered level of consciousness persisting for >24 hours, plus ≥ 2 of the following: fever (>38°C), seizure, focal central nervous system findings, CSF pleocytosis (>5 × 10^6^ cells per L), electroencephalogram (EEG) abnormalities, or diagnostic imaging abnormalities. EEG abnormalities included generalized background slowing and periodic lateralizing epileptiform discharges (PLEDS) and neuroimaging abnormalities included localized edema, mass effect, high/low-density lesions, or infarction and hemorrhage, with localization to the limbic, parietal, frontal, occipital lobes or thalami [1].

**Figure 1:**
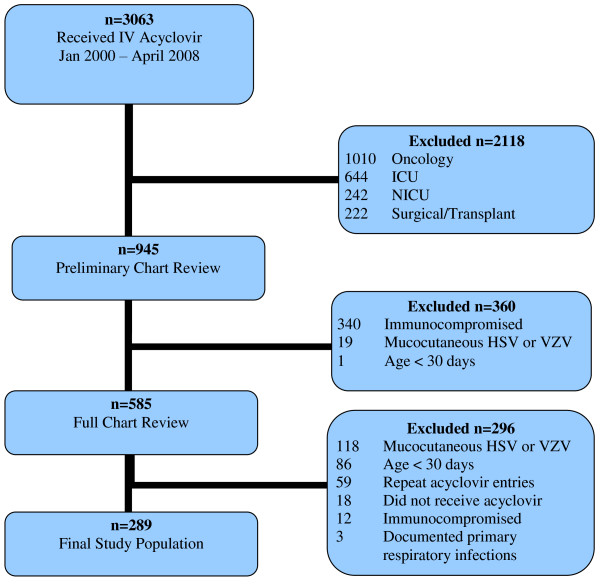
Selection process for study inclusion.

For the current study, children were eligible for inclusion if they had been assessed in our ED, underwent a lumbar puncture, CSF was sent for HSV PCR, empiric acyclovir had been initiated (at least one dose received), and the child was admitted to the PMIU and attended by a Pediatric Hospitalist. This sequence of assessment, testing and treatment was considered a proxy for a physician’s decision to consider the diagnosis of HSE. The study period was January 2000 to April 2008 (January 2000 was selected since this was the date from which electronic charts were available for review). Exclusion criteria were direct admission to the Pediatric Intensive Care Unit (PICU) or a subspecialty inpatient unit, age less than 30 days, immunocompromised state, primary respiratory infection with no neurologic presentation, suspected or confirmed HSV disease restricted to mucocutaneous sites or if varicella zoster virus was the cause of the child’s illness (see Figure 1). At our institution, there is a high threshold for admitting patients with suspected encephalitis to the PICU, representing children with more severe neurologic symptoms and a higher pre-test probability of CNS infection, leading to different physician approaches to clinical decision making.

The primary source for identifying eligible children was the hospital pharmacy database through which we identified all children receiving at least one dose of acyclovir. The encephalitis registry database was used as a secondary source to cross-check identification of children with confirmed HSE who received intravenous acyclovir. One child was identified from the encephalitis database that was not noted in the pharmacy database.

### Study definitions

Cases were defined as children prescribed 14 days or more of intravenous acyclovir and controls as children prescribed a shorter duration. At our institution, a complete course of acyclovir was considered 14 days prior to 1999 and 21 days after 1999 [1]. While physician’s written discharge diagnosis is prone to error and variation in documentation, the prescription of a complete course of acyclovir is an explicit measure of the attending physician’s decision making.

Based on findings of the prospective study of children with HSE [1], the study team generated a number of potential predictors of acute CNS infection, including history, symptoms and physical examination findings at initial assessment, and initial laboratory investigations. A normal CSF WBC count was defined as 0–5 cells ×10^6^/L; adjustment for a bloody tap was performed according to the following formula: true WBC in CSF = actual WBC count in CSF – {(WBC in blood X RBC in CSF)/RBC in blood} [13,14]. Diagnostic investigations completed beyond the first 12 hours of initial assessment (EEG, CT, MRI, CSF PCR) were included in the secondary analysis. DMK reviewed the pharmacy database and encephalitis registry to determine eligibility. Data were abstracted from hospital records by DMK and MY using a standardized data collection form.

Standard statistical methods were employed. Group comparisons were done using the t-test for continuous variables and chi-square for categorical variables; odds ratios and 95% confidence intervals were calculated.

## Results

A total of 3063 patients were prescribed at least one dose of intravenous acyclovir between January 2000 and April 2008. 2774 were excluded for the following reasons: (a) admission to an inpatient unit other than the PMIU (oncology unit n = 1010; PICU n = 644; NICU n = 242; surgical or transplant unit n = 222); (b) suppressed immune system (n = 352); (c) mucocutaneous lesions consistent with varicella zoster virus infection, HSV stomatitis or eczema herpeticum (n = 137); (d) primary respiratory illness consistent with pneumonia or pneumonitis (n = 3); (e) age <30 days (n = 87), (f) repeat or incorrect entries (n = 77) (see Figure 1).

The remaining 289 subjects were included in the analysis; of these, 30 (10%, 95% Confidence Interval 7-13%) were prescribed 14 or more days of intravenous acyclovir (median 20.0, IQR 5.25 days) and 259 (90%, 95% Confidence Interval 87-93%) less than 14 days of such therapy (median 2.0, IQR 2.0 days). All of the children who received acyclovir had CSF HSV testing performed (PCR or culture).

The most common presenting clinical manifestations observed in the 289 included subjects were fever (66%), seizures (46%) and headache (27%). Other manifestations included altered level of consciousness (19%), meningismus (16%), vomiting (10%), ataxia (9%) and personality changes (7%). Intravenous acyclovir was initiated within 24 hours of admission in 95% of cases. Ceftriaxone, cefotaxime and vancomycin were given to 191 (66%), 48 (17%) and 95 (33%), respectively, pending CSF and blood culture results. Anticonvulsants, predominantly benzodiazepines (n = 70, 24%), phenobarbital (n = 47, 16%) and phenytoin (n = 43,15%), were used in 105 (36%).

A comparison of clinical and laboratory variables in children prescribed an incomplete or a complete course of acyclovir is shown in the Table 1. Patients prescribed <14 days of acyclovir received a median of two days of therapy. Patients prescribed ≥ 14 days of acyclovir were likely to have a discharge diagnosis consistent with the treating physicians’ suspicion of acute CNS infection. Twelve of 30 patients received discharge diagnoses of “encephalitis” or “rule out encephalitis”. Other discharge diagnoses included: acute disseminated encephalomyelitis (7/30), headache/migraine (2/30), meningitis/possible meningitis (6/30), seizures (4/30), cerebellitis (3/30), neurologic symptoms not yet diagnosed (1/30) and encephalopathy (1/30). Some patients received greater than one discharge diagnosis.

**Table 1:** Clinical, laboratory and diagnostic investigations of patients who received <14 days and ≥ 14 days of acyclovir therapy

	**<14 days (n=259)**	**≥14 days (n=30)**	**Odds ratio (95% CI)**	**p value**
**Clinical variables, history**				
**Age (years), median (range)**	3.8 (0.01-18)	6.8 (0.07 – 16.7)		0.2
**Sex (male), n (%)**	152 (59)	13 (43)	0.5 (0.2 – 1.2)	0.1
**History of fever, n (%)**	171 (66)	19 (63)	0.9 (0.4-2.0)	0.8
**History of seizure, n (%)**	120 (46)	13 (43)	0.9 (0.4-1.9)	0.8
**History of headache, n (%)**	70 (27)	9 (30)	1.1 (0.5-2.6)	0.7
**History of mucocutaneous HSV, n (%)**	3 (0.8)	3 (10)	14.3 (2.3 – 89)	<0.01*
**Clinical variables, physical examination**				
**Fever (≥38 C), n (%)**	95 (37)	12 (40)	1.2 (0.5 – 2.5)	0.7
**Glasgow Coma Scale ≤ 13, n (%)**	80 (32)	16 (53)	2.5 (1.2-5.3)	0.02*
**Focal neurologic findings, n (%)**	30 (12)	11 (37)	4.4 (1.9-10.2)	0.001*
**Seizure in ED, n (%)**	38 (15)	5 (17)	1.2 (0.4 – 3.2)	0.7
**Initial laboratory measures**				
**WBC, mean (±SD)**	13.1 (7.9)	12.7 (6.3)		0.8
**CSF corrected WBC, median (range)**	4 (0-838)	16 (0-650)		0.05*
**CSF protein, median (range)**	0.3 (0.1-830)	0.4 (0.1-44)		0.8
**Diagnostic investigations**				
**CT head abnormal, n/N completed (%)**	33/194 (17)	7/17 (41)	3.4 (1.2-9.6)	0.03*
**MRI head abnormal, n/N completed (%)**	46/98 (47)	17/24 (71)	2.7 (1.0-7.2)	0.04*
**EEG abnormal, n/N completed (%)**	52/128 (41)	16/22 (73)	3.9 (1.4-10.6)	0.005*

In the primary analysis of predictors at the time of initial assessment, a history of concurrent mucocutaneous HSV infection in the child (p < 0.01), Glasgow Coma Scale (a measure of decreased level of consciousness) ≤ 13 (p = 0.02), focal neurologic findings (p = 0.001) and elevated CSF WBC (p = 0.05) all differed significantly between the two groups. Variables that were not significantly different included: history of fever, seizure, or headache; fever or seizure on initial physical examination; serum WBC and CSF protein (see Table 1). Additional variables not shown in the Table 1 that were not found to be significantly different between the two groups included vomiting, dizziness, behavior changes, dysarthria, ataxia, hallucinations, peripheral band count, CSF glucose, liver function tests and creatinine. No children developed renal failure (>50% rise in baseline creatinine).

Some, but not all, children had additional diagnostic investigations which were not considered part of the child’s initial assessment and were included in the secondary analysis (see Table 1). Compared with children prescribed an incomplete course of acyclovir, children with a complete course of acyclovir more frequently had an abnormal CT head scan (p = 0.03), MRI brain scan (p = 0.04), or EEG (p = 0.005) within the first 48 hours of presentation.

In four of the 289 included patients, HSV-1 was detected in the CSF by PCR. Two had decreased level of consciousness (GCS ≤ 13) and focal neurologic deficits. None had significantly abnormal CSF counts, protein or glucose levels. The EEG was abnormal in 2 of 3 patients tested. Two patients had CT scans of the head, one of which was abnormal. MRI of the brain was abnormal in the 3 patients assessed. All four were prescribed ≥14 days of acyclovir treatment, though one patient received the first acyclovir dose after 24 hours of admission.

## Discussion

We have demonstrated that at our institution 90% of children who received a lumbar puncture, CSF testing for HSV PCR, initiation of empiric intravenous acyclovir and admission to hospital did not complete a full course of acyclovir. These children were hospitalized with a median duration of therapy of two days and more than 70% received additional investigations including EEG and neuro-imaging. Thus, for every one child for whom this sequence of testing and treatment was necessary, nine children received the same sequence unnecessarily.

This study was undertaken from the perspective of the hospital physician who is responsible for making decisions regarding the duration of acyclovir treatment, need for subspecialty consultation and additional investigations, and time of hospital discharge. Hospital physicians recognize that testing, treatment and hospitalization are burdensome to children and families, and to the health care system; further, hospital physicians are aware that acyclovir is nephrotoxic, may lead to acute renal failure and that concurrent use of antibiotics (including ceftriaxone, aminoglycosides and vancomycin), as occurred in the majority of our study patients, may augment the nephrotoxic effects of acyclovir [15-19]. Although HSE is associated with high mortality and morbidity, we believe that the current rate of unnecessary testing and treatment should be considered excessive, and this should motivate further research.

The prospective study by Elbers and colleagues demonstrated that HSE is a rare condition, even in a referral center, with 16 confirmed cases over 12 years, approximately 1–2 cases per year [1]. These investigators identified the following clinical variables and their frequency in the 16 confirmed cases: encephalopathy (defined as depressed or altered level of consciousness persisting for > 24 hours) in 100% (this was a requirement for enrollment in their registry); fever (100%); focal seizures at presentation (69%); history of, or exposure to HSV (50%); hemiparesis (31%); dysphasia (13%). Initial laboratory variables were as follows: CSF pleocytosis (>5 × 10^6^ cells per L) (94%); elevated CSF protein (50%); CSF red blood cells of 50 to 100 × 106 per L (19%). These initial clinical and laboratory variables should inform clinicians however, without a control group (for example, children undergoing a lumbar puncture who do not have HSE), the strength of these variables to predict HSE remains unknown.

Considering the findings from the prospective study and our case–control study together suggests two interpretations which warrant further investigation. First, current practice is not consistent with what is currently known about the clinical and laboratory findings in children with HSE. For example, from the prospective study all children with confirmed HSE had altered level of consciousness and fever and almost all had CSF pleocytosis; in contrast in our case–control study less than 40% had these features. Second, while awaiting a controlled prospective study of children with and without HSE, there are several variables that were associated with HSE in both the uncontrolled prospective study and our retrospective case–control study. These may guide clinicians in the judicious use of CSF HSV PCR testing and initiation of empiric acyclovir.

An important body of literature has emerged regarding testing for HSV in neonates and young infants less than 3 months of age in Emergency Department (ED) settings [9-11]. This research has been stimulated by the lack of consensus regarding which infants merit testing and concerns regarding over-testing. These studies have concluded that factors currently used by physicians in the decision to order CSF HSV PCR testing do not best reflect the likelihood of HSV infection as described by the literature [9]; there is considerable practice variation amongst Emergency Department physicians [10]; and infants undergoing testing have a significantly longer length of hospital stay and higher hospital charges [11]. The results of our case–control study of all-aged children in an Inpatient Pediatric setting have identified remarkably similar findings. We have further highlighted the downstream impact of testing upon hospital length of stay, treatment with intravenous acyclovir, ordering of advanced investigations and requesting of subspecialty consultation.

Shah and colleagues have suggested that both the direct and indirect costs of testing are substantial, arguing for the development of a clinical prediction rule to identify low-risk infants [11]. We strongly concur that derivation of a clinical prediction rule is warranted, and suggest that such a rule include all-aged children, perhaps with stratification in two age groups (< 3 months; > 3 months). Clinical prediction rules aim to reduce uncertainty, improve accuracy in medical decision making and minimize the use of potentially harmful diagnostic tests.

They have been defined as clinical decision-making tools that quantify the relative importance of three or more variables from history, physical examination, or simple tests to provide the probability of an outcome or suggest a single diagnostic or therapeutic course of action for an individual patient [20,21].

Similar to the studies in infants < 3 months [9-11], the primary limitation of this study was its retrospective nature. The use of retrospective data is limited by the accuracy and completeness of the original data collection. Our study included only children admitted to the PMIU, and is therefore not generalizable to children with more severe disease, such as those admitted to intensive care units. Clinical decision making varies by practice setting where there are differences in pre-test probabilities and variations in practice. While it is possible that some children were discharged from our institution after cessation of acyclovir therapy only to be diagnosed with HSE at another institution, this is unlikely. Our institution is the only regional quaternary children’s hospital, and other hospitals commonly refer children with chronic and life threatening illnesses such as encephalitis to our center.

## Conclusion

Our study demonstrates that a large proportion of children who receive a lumbar puncture, CSF testing for HSV PCR and initiation of empiric intravenous acyclovir do not complete a full course of such therapy. As shown for neonates and young infants, there is significant physician practice variation; current practice does not reflect what is currently known; and over-use of testing and treatment is burdensome to children, families and the health care system. Derivation of a clinical prediction rule identifying all-aged children at low risk for HSE is warranted. Variables which may be predictive include altered level of consciousness, focal neurologic findings and CSF pleocytosis. Future studies are recommended to determine if these variables remain predictive in other populations of infants at risk for HSE.

## Abbreviations

HSE: Herpes simplex encephalitis; OR: Odds ratio; CI: Confidence interval; PCR: Polymerase chain reaction; HSV: Herpes simpex virus; LOC: Level of consciousness; MRI: Magnetic resonance imaging; FLAIR: Fluid attenuation inversion recovery; CSF: Cerebral spinal fluid; PMIU: Pediatric medicine inpatient unit; PICU: Pediatric intensive care unit; NICU: Neonatal intensive care unit; CT: Computed tomography; CBC: Complete blood count; EEG: Electroencephalogram; GCS: Glasgow Coma Scale; IQR: Interquartile range; LP: Lumbar puncture.

## Competing interests

The authors have no competing interests to disclose.

## Authors’ contributions

PCP conceived of the study. DMK and PCP designed the study. DMK and MY performed the chart review. DMK, MM, AB and PCP analyzed the data. DMK drafted the manuscript and all authors read and approved of the manuscript.
